# IUC26692-90 Benchmarking Artificial Intelligence-Based Clinical Prediction Models for Immune Checkpoint Inhibitor Response and Overall Survival in Bladder Cancer

**DOI:** 10.1093/oncolo/oyag286.008

**Published:** 2026-08-21

**Authors:** R Das, B Gonsalves, V Ravindran, Y Agarwala, D Khanna, A Tiwari, A Ghose, G Luigi Banna, R Dylan Frazer, S Adeleke, A Maniam

**Affiliations:** Faculty of Life Sciences and Medicine, King's College London, London - United Kingdom; Faculty of Life Sciences and Medicine, King's College London, London - United Kingdom; UCL Cancer Institute, London - United Kingdom; Faculty of Medicine, Imperial College London, London - United Kingdom; University of Buckingham Medical School, Buckingham - United Kingdom; Department of Oncology, Princess Alexandra Hospital NHS Trust, Harlow - United Kingdom; Cancer Division, University College London, London - United Kingdom; Department of Medical Oncology, Portsmouth Hospitals University NHS Trust, Portsmouth - United Kingdom; Velindre Cancer Centre, Cardiff - United Kingdom; Curenetics, London - United Kingdom; Department of Medical Oncology, Portsmouth Hospitals University NHS Trust, Portsmouth - United Kingdom

## Abstract

**Background:**

Bladder cancer (BLCA) is a common urological malignancy with high recurrence and heterogeneous treatment response. Immune checkpoint inhibitors (ICIs) have transformed advanced BLCA treatment, yet response prediction remains challenging. AI-based models have been developed to predict ICI response and OS prognosis in BLCA. To benchmark AI models predicting ICI response and overall survival (OS) in BLCA, evaluating both predictive performance and reporting quality.

**Methods:**

A search of OVID MEDLINE and EMBASE yielded 1412 raw records; 281 full-text articles were screened, of which 13 BLCA-specific studies met eligibility, 8 contributing quantifiable AUCs to the synthesis. AI-plus-clinical or combined-AI AUCs were excluded, retaining single-AI-model AUCs only. Studies were categorised into Category A (ICI response, RECIST-defined) and Category B (OS prognosis). As few studies reported confidence intervals, meta-analysis was not feasible; descriptive synthesis (medians, ranges) was performed instead.

**Results:**

Category A comprised 7 AUCs from 4 studies (cohorts 11–384 patients); 6 of 7 were derived from IMvigor210, indicating overlap between studies, while the smallest-n estimate (n  =  11) came from a separate cohort (GSE111636). The most robust model (Chen S *et al.* 2020, 10-gene ICIR-Score) achieved an internal validation AUC of 0.752 (95% CI 0.654–0.834, n  =  98). External validation AUCs ranged 0.640–0.733 (median 0.669); whole-cohort AUCs ranged 0.719–0.805 (median 0.762). Category B comprised 24 OS AUC estimates from 4 studies. Training AUCs (cohorts 278–405 patients, all TCGA-BLCA-derived) increased across timepoints: 1-year median 0.667, 3-year median 0.695, 5-year median 0.730. External validation came from one study only (Zhang X *et al.* 2025, GSE32548 and GSE32894): 1-year median 0.670, 3-year median 0.710, 5-year median 0.726. Internal validation and whole-cohort estimates were likewise from one study only (Li Z *et al.* 2024, AUCs 0.751–0.763).

**Conclusions:**

AI models show moderate-to-good discrimination for ICI response and OS prognosis in BLCA, but evidence is constrained by reliance on a single trial cohort (IMvigor210) and training cohort (TCGA-BLCA), with internal and external OS validation each corroborated by only one study, limiting generalisability. Larger, prospective, externally validated studies with consistent uncertainty reporting are needed.

